# Editorial

**Published:** 1844-06-29

**Authors:** 


### THE MEDICAL EXAMINER.

PHILADELPHIA, JUNE 29, 1844.

### THE AUTOMATON SPEAKER.

Our attention was first directed to this triumph of human ingenuity by the Editor of the New York Journal of Medicine, who described it as the result of eighteen years unceasing labour, by a person named Faber. "It is constructed upon the model of the human organs of voice,—the tongue, larynx, &c., being made of caoutchouc. * * * The automaton is represented by a bearded Turk, and the articulations are produced by playing upon sixteen keys. We were quite surprised at being addressed by the automaton, in words very distinctly articulated, thus: 'Welcome, Doc-tor For-ry; please ex-cuse my slow e-nun-ci-a-tion.' After giving various other illustrations of his vocal powers, the automaton sang 'Hail Columbia, &c.' As we were about leaving he said, 'gen-tle-men, I thank you for your vis-it.'"

And after this detail so deeply interesting to the physiologist, the editor adds: "But after all, *cui est bono?*" Could there be a case in which the cry of *cui bono* could be more inappropriate! The problem of the mechanism of the human voice has been, and is, one of the most deeply interesting to the physiologist, and the *physicien*; and even at the present day speculation upon speculation is hazarded in regard to it. The wisest heads and the most expert hands, separately and combined, have been busied with attempts at discovering the mode in which articulated voice is effected; fruitlessly, however, until this ingenious mechanic succeeded—after having spent the best part of a life of indefatigable exertion—in accomplishing that on which Von Kempelen and every other mechanician had failed. A friend of our own, well versed in the mechanic arts both scientifically and practically, after having bestowed unwearied and protracted attention on the formation of a speaking machine, succeeded at length in constructing one that imperfectly enunciated the vowel-sounds and one or two simple consonants, as in mamma and papa, but he could proceed no further. Yet here we had a machine, in which the complicated problem could have been completely studied, and in which the different portions of the larynx and vocal tube were thrown into action as they are in man, and admitted of close and intimate inspection: the key, in fact, to the solution of a most difficult physiological problem is discovered, and it is gravely asked, "*cui* est *bono?*" Still the question does not excite in us so much surprise when we read the following observation:

"As voice," he says, "is the sound produced by air drawn from the lungs through the larynx, causing a vibration of the chordæ vocales, it is a function of *animal* life; but the function in the animals inferior to man, as well as in the idiot, is limited to the production of the *simple* or *instinctive* voice; whilst in intellectual man, it becomes sufficiently complicated for the purpose of articulation. This is regarded as an evidence of man's intellectual superiority. Here, however, we find the same phenomena produced by an apparatus of caoutchouc, and a bellows!"

And what otherwise could be expected. The organ of voice exists in the idiot as in the individual possessed of his full intellectual powers; but it is useless in the former, in consequence of his not having an intellect to guide it. In like manner, in the automaton we have the organ, which is equally useless, until thrown into action under a properly regulated volition.

The sad part of the story remains, however, to be told. When the ingenious inventor of this automaton was about to reap the reward of his toil and ingenuity; and when the scientific of this city—of whom there are many; and the curious—of whom there are more, were full of the expectation of deriving an amount of positive knowledge in regard to the mechanism of the voice not previously within their reach,—under some momentary excitement, the offspring was destroyed by its unhappy parent, and not a fragment now remains of the unrequited toil of so many years of successful experiment. Benevolent individuals have, indeed, stepped forward with offers of pecuniary aid towards the fabrication of another; but at present, we regret to say, with very slight prospect of success.

### NEW ORLEANS MEDICAL JOURNAL.

We have received the first number of a new Medical Journal, published in the city of New Orleans, under the Editorial care of Erasmus D. Fenner, M. D., and A. Hester, M. D. It is to contain about one-hundred and twenty pages, 8vo., and to be issued "bi-monthly," at five dollars per annum. In an "introductory address," not remarkable for its brevity, the Editors speak of the inducements, the objects and the aspirations with which they have undertaken the task.

"What," they exclaim, "is the humiliating declaration we are bound to make! Few—but very few Physicians in the South have ever offered contributions to medical literature; and there is not a Medical Journal to be found in the United States south of Louisville. Will it be believed abroad when we add, that in this vast and interesting region, there exists no less than four Medical Colleges, whose halls are annually attended by students, and which are granting Diplomas from year to year?" Four Medical Colleges granting degrees and not one Journal! That is indeed too bad, and we think Drs. Fenner and Hester are entitled to the support not only of the "Medical Colleges," but of the profession of that region in their laudable efforts to supply the deficiency. New Orleans is a large city, having extensive commercial relations with the surrounding country, as well as the remoter parts of the United States and Europe, and we can conceive of no possible reason why a good Medical Journal should not be sustained there. The bright side of the picture presented by our new "confrères," is exhibited in the following language:

"We even indulge the hope that in a short time we shall see New Orleans, the Emporium of a vast and varied commerce as it is, become also a *focus* to which shall be concentrated the rays of medical light from all parts of the world, again to be disseminated for the most useful purposes." Our enthusiasm, we confess, has been checked by the lapse of too many years to allow us to *hope* with the Editors for the fulfilment of those bright visions "in a short time," but they have our best *wishes* for their attainment.

There is one position assumed and sought to be maintained in the address, which we feel bound to notice, although we do it with regret, as we by no means wish to engage in controversy with any of our bretheren of the press, and especially those who are just entering into the brotherhood; but the question involved is one of importance, and ought to be put at rest. "We," say the editors, "have been raised in the south-west; our professional career has been chiefly in the South, and we can assert an experience of fifteen years in its peculiar maladies. We therefore have a right to declare that the diseases of the South can only be studied and learned in the South."

"The elements of the profession," they say "may be studied to perfection in the Capitols of Europe and the United States," but the peculiar diseases of the south and west, can only be treated by a physician who, after commencing "his observations *de novo*," has established "for himself a new code of principles and practice." "On this point we feel confident we are expressing an opinion almost universally entertained in the south; for often have we heard it deliberately remarked by intelligent physicians, that a patient attacked by congestive fever in the form often witnessed on the banks of the Yazoo or Red River, would be much more safe under the management of some intelligent Planter or Overseer who had long resided in this region, and who was perfectly familiar with the disease, than he would be in the hands of the ablest physicians of London or Paris, who had never practised beyond their precincts, and who would be guided in his treatment solely by the general principles of medicine."

This, we are aware, is but the echo of an assertion oft repeated by a prominent teacher in the south-west, the origin and objects of which, however, are perfectly well understood. If the south have *peculiar* diseases which can only be *studied* in the south, it is equally so of the north, of the west, of the east, of the mountains and of the vallies! A man who studies his profession ever so profoundly in one place, is totally unfit to practice it in another! The "planters" and "overseers," and of course the mechanics, the grandmothers and old nurses of one locality, are better qualified to prescribe for the sick of the place than the best educated physician who may have mastered his profession in Philadelphia, in Edinburgh, in London, or in Paris! If this be true, what becomes of the science? Diseases must be regarded as entities, all remedies as specifics, and those who prescribe them—as empirics! This degrading doctrine has been most industriously promulgated in the south-west for years past, and what has been the result of such enlightened teaching? Since the establishment of Medical Schools in the Valley of the Mississippi, comparatively few young men from that quarter seek instruction elsewhere, under the belief that there only can they learn the peculiar diseases of the South. What the effect has been, let our new cotemporaries, whose "professional career has been chiefly in the south," answer: "Who does not perceive that the Medical Profession has been for sometime gradually losing caste and respectability in the south—that unworthy and incompetent members are constantly gaining admission into its ranks—and that the Charlatan and Empiric annually find it less difficult to maintain a successful competition with the licensed Practitioner?"

With how much more success the diseases of the south are studied by those who live there, the following facts will show. The first article in the Journal is "an Essay on Yellow Fever, by J. F. Beugnot, D. M. P., read before the Louisiana Medico-Chirurgical Society, Sept. 1843." It commences thus: "Mr. President and Gentlemen,—Since I begun the practice of medicine in New-Orleans, I have often been astonished with one fact which has doubtless struck you as well as myself; I allude here to the *diversity of opinions among Medical Gentlemen on the subject of Yellow-Fever, and in regard to every thing connected with it.*" The views of that disease put forth by Dr. Beugnot, the Editors testify, are concurred in by "some of the ablest Physicians of New Orleans;" but are "condemned by a large and respectable class." The diametrically opposite treatment of cases in the Charity Hospital of N. O., reported by Dr. Slade, utters the same language. After all, it will be found that those who *practise* with most credit to themselves and advantage to their patients, *in all parts of the world*, are they who are best acquainted with the principles of pathology and therapeutics—in other words, those who have most thoroughly studied medicine as *a science.* English, French, and American naval surgeons, find themselves at no loss in treating the diseases of those under their charge, in any country or climate where their official duty calls them. Well established principles are of general application; and those who are truly instructed in the philosophy of our science, find little difficulty in the proper application of its principles to all the modifications of disease whenever and wherever found.

Having expressed our opinion somewhat in opposition to that of the Editors on this point, we hesitate not to welcome this new auxiliary in the work of diffusing medical knowledge. It comes from an interesting section of the country with which we desire to become better acquainted.—The specimen of the Journal before us contains a large amount of interesting matter, contributed by some excellent members of the profession; and if the future numbers shall continue to be enriched by their communications, and shall contain an equal amount of valuable matter, it must receive, as it will certainly deserve, the encouragement and support of the profession.

---

### TRANSYLVANIA UNIVERSITY.

Sometime since it was announced in the Journals that Professors Bartlett and Cross had resigned the chairs respectively occupied by them in this Institution. From the annual circular which we have since received, it appears that the Faculty has been reorganized, and that Dr. Lotan G. Watson, of North Carolina, and Dr. Leonidas M. Lawson, of Cincinnati, have been elected to supply the places of Drs. Bartlett and Cross—the former as Professor of Theory and Practice, and the latter as Professor of General and Pathological Anatomy and Physiology.

By the new arrangement, Dr. Dudley retains the chair of Surgery, but relinquishes the department of Anatomy, to which his late assistant, Dr. James M. Bush, has been appointed.

